# Food Insecurity and Gastrointestinal Symptom Burden in Adults with Celiac Disease: A Cross-Sectional Study

**DOI:** 10.3390/nu18050873

**Published:** 2026-03-09

**Authors:** Katrina S. Rbeiz, Madison A. Hooper, Hana F. Zickgraf, Alyson Basil, Joanna Perl, Dawn W. Adams

**Affiliations:** 1 Department of Psychological Sciences, Vanderbilt University, Nashville, TN 37235, USA; madison.a.wagener@vanderbilt.edu; 2 Center for Human Nutrition, Vanderbilt University Medical Center, Nashville, TN 37232, USA; hana.zickgraf@vumc.org (H.F.Z.); alie.basil@vumc.org (A.B.); joanna.s.perl@vumc.org (J.P.); dawn.w.adams@vumc.org (D.W.A.)

**Keywords:** celiac disease, food insecurity, gluten-free diet, gastrointestinal symptoms

## Abstract

**Background/Objectives:** Food insecurity (FI) is associated with adverse physical and mental health outcomes and poses unique challenges for individuals with celiac disease (CD), who must adhere to a lifelong, strict gluten-free diet (GFD) as their sole treatment. Gluten-free foods are often more expensive and less accessible, potentially exacerbating dietary burden and symptom severity. This study aimed to examine associations between food insecurity and gastrointestinal symptoms, dietary burden, disordered eating symptoms, and mental health outcomes in adults with CD. **Methods**: Participants were 397 adults with CD who completed a routine pre-visit screener at a tertiary celiac disease clinic in the southeastern United States. Food insecurity was assessed using the Hunger Vital Signs. Measures included gastrointestinal symptoms (GSRS), impact of following a gluten-free diet (IGFDQ), avoidant/restrictive eating (NIAS), eating disorder psychopathology (EDE-Q7), anxiety (GAD-2), and depression (PHQ-2). Group comparisons between food-secure and participants screening positive for food insecurity (high-risk of FI) were conducted using independent *t*-tests or Mann–Whitney U tests as appropriate. Missing data were handled using multiple imputations. **Results**: A total of 15.6% of participants were at high risk of FI. Compared to food-secure individuals, participants at high-risk of FI reported significantly greater gastrointestinal symptom severity, including bloating, constipation, diarrhea, and overall symptom burden. Participants at high risk of FI also reported significantly greater perceived burden of adhering to a gluten-free diet. Additionally, higher levels of avoidant/restrictive eating symptoms and shape/weight overvaluation were observed among high-risk FI individuals, though group differences in anxiety and depression symptoms were not statistically significant. **Conclusions**: FI is associated with greater gastrointestinal symptom burden and increased difficulty adhering to a gluten-free diet among adults with celiac disease. These findings highlight risk of FI as a clinically relevant barrier to effective disease management and underscore the importance of routine screening and targeted support for high-risk FI individuals with CD.

## 1. Introduction

Food insecurity (FI) is defined as inadequate or inconsistent access to safe and nutritious food as a result of inadequate resources (e.g., money) to support an active and healthy lifestyle [1,2,3]. In 2023, the United States Department of Agriculture estimated that 13.5% of households in the United States qualified as food insecure. The food security of a household exists on a continuum ranging from high food security, defined as consistent access to nutritious food, to very low food security characterized by reduced food intake and significant disruptions in eating patterns among one or more household members [4]. FI is associated with a broad range of adverse physical and mental health outcomes. Specifically, individuals experiencing FI are more likely to develop chronic health conditions, such as diabetes and cardiovascular disease, as well as mental health concerns, including depression, anxiety, sleep disturbances, and disordered eating [1,5,6,7]. Prior research has also found that exposure to FI is associated with low diet quality, including fewer whole grains, more solid fats, and added sugars [8]. Despite these poor health outcomes, Park and colleagues found that FI was associated with lower access to care, including delay in receiving necessary medical care and delay in obtaining necessary prescription drugs [9].

Celiac disease (CD) is a serious autoimmune enteropathy triggered in genetically susceptible people by ingestion of the protein in wheat, barley, and rye called gluten. More than 20 parts per million (ppm), roughly the size of the point of a pen, of gluten is enough to trigger a response in a person with CD [10]. Without treatment, these people develop inflammation of the small intestine, leading to malabsorption of micro- and macro-nutrients and associated conditions like osteoporosis, anemia, as well as neurological and reproductive problems, and an increased risk of malignancy [11]. At this time, the only modality for disease management is a lifelong adherence to an entirely gluten-free diet (GFD). Despite its positive impact on physical health and quality of life, 11–44% of CD patients nevertheless also report negative impacts of adherence to GFD on quality of life and social-emotional wellbeing [12,13,14,15,16,17]. The higher cost of gluten-free products is a commonly cited negative impact of the GFD [18,19].

CD patients who report struggling with GFD adherence are at elevated risk for anxiety, depression, poor general health, and active CD symptoms compared to other CD patients [20,21,22]. This is striking given that CD patients in general appear to experience higher rates of psychopathology compared to the general population [23,24,25]. Whereas the relationship between CD and affective symptoms may be mostly accounted for by disease impact and non-specific [23,24], there is a bidirectional link between CD and disordered eating, suggestive of disease-specific risk factors [26]. While autoimmunity in general is associated with disordered eating [27], CD is relatively unique among chronic illnesses in the need for permanent and exacting avoidance of a common food ingredient [28]. The need to maintain a GFD may be a CD-specific risk factor for developing disordered eating [28,29,30,31].

In the case of CD, there is no inherent pharmacologic or financial assistance for treatment (gluten-free food) in the form of a prescription and/or insurance coverage. The ability to control the disease depends entirely on the patient successfully managing the GFD, a lifestyle-based intervention of high complexity. Unlike other diseases, this places the entire burden of disease management (mental, social, and financial) on the patient. Studying FI in patients with CD is essential due to the unique and often challenging dietary restrictions these individuals must maintain. Ma and colleagues found in a 2022 study of adult CD patients who were experiencing FI that only about 25% of them adopted a GFD compared to about 80% adoption of GFD in CD patients who are food secure [32]. Similar findings were demonstrated in a Canadian study that found a significant linear relationship between FI and the odds of children with CD adhering to a GFD; as the severity of FI went up, so did the odds of noncompliance [33]. Food stability status also impacts the amount and types of foods that patients purchase; as food stability decreases, patients are more likely to alter the type of GF foods that they purchase (from unprocessed to processed) [33]. Similar findings came from a British study looking at the impact on patients with CD after the prescription for gluten-free food was withdrawn. This study noted that patients were more likely to eat less overall, substitute the types of food purchased, and cut back on other expenses as a means to afford gluten-free food after the prescription was withdrawn [34]. Notably, they found an implicit impact on certain patient populations over others, with those less mobile, permanently sick/or disabled, and or having lower incomes affected more by the prescription withdrawal [34]. It has also been found that upwards of 70% of food-insecure CD patients do not meet the recommended daily intake for a variety of essential vitamins and minerals [32]. Further complicating food security for CD patients, existing sources of food assistance like food pantries rarely stock gluten-free alternative products. This represents a significant gap in accessibility to treatment for people of lower economic status.

Furthermore, FI in CD patients intersects meaningfully with mental health outcomes. Individuals with CD exhibit elevated rates of anxiety, depression, disordered eating behaviors, and FI, all of which are independently associated with greater difficulty adhering to a GFD. These factors also contribute to a perceived negative impact of the diet, creating a feedback loop that increases both psychological distress and the risk of physical complications. The negative experience of adhering to a GFD, therefore, emerges as a promising target for psychosocial, nutritional, and financial interventions aimed at improving quality of life and health outcomes in this population. Addressing FI may not only facilitate better dietary adherence but also reduce the broader mental and physical health burden associated with CD. Despite these overlapping risks, no study to date has examined how FI interacts with disordered eating, mental health, and dietary burden specifically within individuals with CD, an already at-risk population due to the lifelong requirement of strict gluten avoidance. Building on prior research, it is plausible to hypothesize that FI will be significantly associated with eating disorder symptoms, including Avoidant/Restrictive Food Intake Disorder (ARFID), features, and broader disordered eating, as well as with depression, anxiety, and the impact of a GFD. Individuals with CD and those experiencing FI are both at increased risk of adverse mental health outcomes, including depression and anxiety, largely attributable to the psychosocial stress and adversity associated with these conditions rather than disease-specific mechanisms [35,36]. Beyond general psychological distress, both CD and FI have also been linked to disordered eating behaviors, with emerging evidence suggesting causal pathways shaped by restrictive dietary demands, concerns about food safety or adequacy, and heightened vigilance around food choices [37].

We hypothesized that adults with celiac disease who screened positive for risk of FI would report greater gastrointestinal symptom burden and greater perceived burden of adhering to a gluten-free diet compared to food-secure individuals. We further hypothesized that risk of FI would be associated with higher levels of avoidant/restrictive eating symptoms and eating disorder psychopathology. Given mixed findings in prior literature, associations with anxiety and depressive symptoms were examined exploratorily.

## 2. Materials and Methods

### 2.1. Participants

The naturalistic clinical dataset contained 732 responses from 421 unique participants who completed a universal pre-visit screener as part of routine assessments conducted at a medical center. The universal screener was implemented within the context of a larger IRB-approved study examining eating-related symptoms in gastroenterology patients; however, the present manuscript represents a cross-sectional secondary analysis of screener data and does not evaluate intervention outcomes. When a single patient completed the screener multiple times, the earliest recorded response was retained. Of these, 397 participants had been diagnosed with CD and were included in the analysis. The final sample consisted solely of individuals with CD, as the focus of the study was to examine FI and related factors specifically in this population. 

Participants were established adult patients of a tertiary celiac disease clinic with a documented diagnosis of CD in the electronic medical record. Although diagnosis was self-reported on the survey instrument, inclusion in analyses required a chart-documented diagnosis of CD. No additional exclusion criteria were applied. Participants with comorbid gastrointestinal conditions, psychiatric diagnoses, or eating disorders were not excluded, as the dataset was derived from routine clinical care. Information regarding biopsy confirmation, serologic status, and duration of adherence to a gluten-free diet was not systematically available and therefore not used as eligibility criteria. The data was collected from a CD clinic located in the Southeastern part of the United States. This clinic provides care to roughly 2000 adult patients. The study was conducted in accordance with the Declaration of Helsinki and approved by the Institutional Review Board of Vanderbilt University Medical Center (IRB #230320 and approved on 18 May 2023). Data were collected between May 2023 and February 2025.

### 2.2. Measures

Detailed instrument scoring can be found in Appendix A.

#### 2.2.1. Food Insecurity

Risk of FI was measured using the Hunger Vital Signs assessment [38], a validated screening tool for FI consisting of two questions: (1) “Within the past 12 months, did you worry whether your food would run out before you got money to buy more?” and (2) “Within the past 12 months, did the food you bought just not last and you didn’t have money to get more?” These items were rated on a three-point scale: “never true,” “sometimes true,” and “often true.” Participants were considered to have screened positive for risk of FI if they scored at least a 1 on either of the two questions, reflecting concerns about food availability or insufficient quantity due to financial constraints. 

#### 2.2.2. Celiac Diagnosis

Participants self-reported their CD diagnosis on the survey; however, inclusion in analyses required a documented diagnosis of CD in the electronic medical record.

#### 2.2.3. Avoidant/Restrictive Eating

In the present study, the Nine Item ARFID Screen (NIAS) [39] was used to assess avoidant/restrictive eating patterns. The NIAS is a brief self-report instrument designed to explicitly measure avoidant/restrictive eating associated with the three ARFID presentations described in the DSM-5: appetite, fear, and picky eating. The picky eating subscale measures avoidance of foods based on their sensory properties (e.g., “I dislike most of the foods that other people eat”). The appetite subscale measures a limited interest in eating (e.g., “I’m not very interested in eating; I seem to have a smaller appetite than other people”). The fear subscale measures fear of negative consequences from eating (e.g., “I eat a small portion because I am afraid of GI discomfort, choking, or vomiting”). Each item is rated on a 6-point Likert-type scale: 0 (Strongly disagree), 1 (Disagree), 2 (Slightly disagree), 3 (Slightly agree), 4 (Agree), and 5 (Strongly agree). The three items from each scale are summed to create subscale scores ranging from 0 to 15, with higher scores reflecting higher levels of each driver of restrictive/avoidant eating [39]. The NIAS has exhibited strong evidence of construct, convergent, and external validity in community samples and eating disorder clinic populations [39,40]. 

#### 2.2.4. Eating Disorder Psychopathology

The Eating Disorder Examination-Questionnaire 7 (EDE-Q7) [41] was used to assess weight/shape ED symptoms. The EDE-Q7 comprises 7 items from the EDE-Q [42], a widely used questionnaire assessing the frequency or severity of ED symptoms over the past 4 weeks. The items are rated on a 0 to 6 Likert scale based on either frequency (No days, 1–5 days, 6–12 days, 13–15 days, 16–22 days, 23–27 days, or Every day) or degree (Not at all, Slightly, Moderately, Extremely). The items can be combined to generate three subscale scores (e.g., Dietary Restraint, Shape/Weight Overvaluation, Body Dissatisfaction) and a global score. The global EDE-Q7 score is calculated by averaging the four subscales.

#### 2.2.5. Gastrointestinal Symptoms

The Gastrointestinal Symptom Rating Scale (GSRS) [43] is a self-report instrument designed to evaluate common symptoms of gastrointestinal disorders. The GSRS consists of 15 items, each rated on a seven-point Likert scale ranging from “no discomfort” to “very severe discomfort”. The items can be combined into five symptom clusters reflecting reflux syndrome (e.g., heartburn and acid regurgitation), abdominal pain (e.g., abdominal pain, hunger pains, and nausea), indigestion, diarrhea, and constipation.

#### 2.2.6. Impact of Following a Gluten-Free Diet

The Impact of a Gluten Free Diet Questionnaire (IGFDQ) assesses the burden of a GFD, including limited food choices, interference with social activities, and impact on emotional well-being over the past 7 days [16,44]. Items are rated on a 1 to 5 scale, with 1 representing “no burden or interference” and 5 representing “always a burden or a complete interference.”

#### 2.2.7. Symptoms of Depression and Anxiety

The GAD-2 [45] is a two-item (“feeling nervous, anxious, or on edge” and “not being able to stop or control worrying”) screening measure used to assess core symptoms of generalized anxiety over the past two weeks. Items are rated on a scale of 0 to 3, with 0 being “not at all”, 1 being “several days”, 2 being “more than half the days”, and 3 being “nearly every day”. Total scores on the GAD-2 range from 0 to 6. 

The PHQ-2 [46] is a two-item (“little interest or pleasure in doing things” and “feeling down, depressed, or hopeless”) screening instrument assessing the frequency of depressed mood and anhedonia over the past two weeks. Each item is rated on a 4-point Likert scale, with scores of 3 or higher consistent with elevated risk for depressive disorder. Total scores on the PHQ-2 range from 0 to 6. 

### 2.3. Statistical Methods

Group comparisons were conducted to examine differences between food-secure and high-risk of FI participants on the measures of interest. Independent *t*-tests and Mann–Whitney U tests were used, depending on the distribution and nature of the data for each variable. Effect sizes, specifically Cohen’s d, were calculated to assess the magnitude of differences observed between food-secure and high-risk of FI groups. All analyses were conducted using R version 4.4.1 [47].

To address multiplicity across multiple symptoms and psychosocial outcomes, we prespecified the Gastrointestinal Symptom Rating Scale (GSRS) Total score as the primary outcome reflecting overall gastrointestinal symptom burden. All other outcomes (GSRS subdomains, Impact of a Gluten-Free Diet [IGFD] total score, Nine-Item ARFID Screener [NIAS] subscales and total score, Eating Disorder Examination Questionnaire-7 [EDE-Q7] subscales and global score, GAD-2, and PHQ-2) were considered secondary or exploratory outcomes. The primary outcome was tested at a two-sided α = 0.05. For all non-primary outcomes, we applied the Benjamini–Hochberg false discovery rate (FDR) procedure with q = 0.05 to control for multiple comparisons.

We conducted a multivariable linear regression analysis with GSRS Total as the dependent variable and FI status as the primary predictor, adjusting for basic demographic factors (age and sex). These covariates were selected a priori based on their potential association with both FI risk and gastrointestinal symptom reporting. Covariate selection was based on conceptual relevance and prior literature rather than statistical significance in univariate analyses to avoid data-driven model specification. Adjusted beta coefficients with 95% confidence intervals (CIs) are reported. The regression model was used to estimate the association between FI risk and gastrointestinal symptom burden rather than to infer causal relationships.

A completed STROBE checklist and detailed instrument scoring procedures are provided in Appendix B (Table A1).

## 3. Results

### 3.1. Descriptive Statistics

A total of 397 participants diagnosed with CD were included in the analysis (Table 1). The prevalence of FI in this sample was 15.6% (N = 62), which is lower than a recent estimate of the overall prevalence in Tennessee (36% of adults in households with children and 17% of adult-only households) [48,49]. CD patients with FI were younger (M = 37.67, SD = 15.65) on average than those without FI (M = 41.77, SD = 17.42). The sample was predominantly female (77%) and White (97%), with no statistically significant differences in age, sex, or race distribution between food-secure and high-risk groups (*p*s > 0.05).

### 3.2. Missing Data

Missing data were handled using the multiple imputation methods implemented in the mice package in R version 4.4.1 [50]. The mice package applies Fully Conditional Specification (FCS), where each incomplete variable is imputed using a separate conditional model. Predictive mean matching (PMM) was used as the imputation method at the item level. PMM was selected because it preserves the distribution of the variables and avoids implausible values [51].

Overall, missingness across study variables was modest (~8%; range = 3–17%). The highest missingness was observed among the GSRS items (15–17%), with Item 3 exhibiting the greatest proportion of missing responses (17%). The proportion of missing data for each item, as well as missingness by food insecurity group, is reported in Table A2. To examine whether missingness differed across food insecurity groups, the total number of missing items per participant was compared using a Welch’s *t*-test. The number of missing responses did not significantly differ between food-secure and food-insecure participants (*p* = 0.075), suggesting that missingness was not systematically associated with food insecurity status. Additionally, sensitivity analyses conducted using complete-case data produced results that were consistent with those obtained using the multiply imputed datasets (see Table A2).

### 3.3. Group Comparisons

To explore the relationship between FI and key psychological and dietary factors, group comparisons were conducted using independent *t*-tests and Mann–Whitney U tests (Table 2).

A multivariable linear regression analysis was conducted to examine the association between FI status and GSRS total while adjusting for age and sex (Table 3). FI status was significantly associated with higher GSRS total scores (β = 0.54, SE = 0.16, 95% CI [0.22, 0.86], *p* = 0.001). Age (β = 0.01, SE = 0.01, 95% CI [−0.01, 0.02], *p* = 0.19) and female sex (β = 0.08, SE = 0.14, 95% CI [−0.20, 0.36], *p* = 0.57) were not significantly associated with GSRS total.

**Figure 1:**
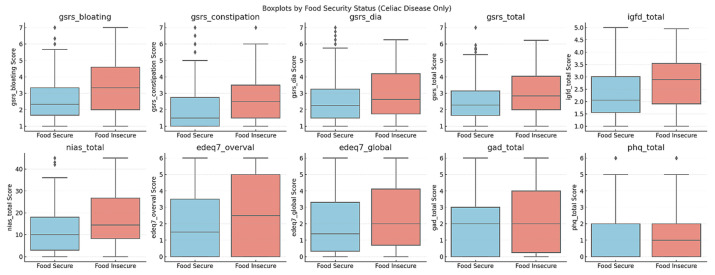
Box plots illustrating differences in psychological and health-related variables between participants who screened positive for risk of food insecurity (FI; *n* = 62) and food-secure participants (*n* = 315). Boxes represent the interquartile range (IQR), horizontal lines indicate medians, and whiskers extend to 1.5 × IQR.

A significant difference emerged in the Impact of Gluten-Free Diet (IGFD total) between food-secure and high-risk of FI groups. Specifically, patients with FI reported more difficulties in adhering to a GFD than their food-secure counterparts. This difference was visually represented in a boxplot (Figure 1) comparing IGFD total scores, which showed a higher distribution of scores among high-risk of FI individuals compared to food-secure participants, indicating that these individuals faced greater challenges in managing their dietary restrictions.

Participants who screened positive for risk of FI reported significantly higher NIAS total scores and higher scores across NIAS subscales (picky eating, appetite/low interest, and fear of aversive consequences) compared to food-secure participants. Differences in EDE-Q7 restraint, GSRS pain, global eating disorder psychopathology, anxiety (GAD-2), and depression (PHQ-2) were not statistically significant.

After applying the Benjamini–Hochberg false discovery rate correction to all non-primary outcomes, the overall pattern of findings was preserved. Differences in IGFD total score, NIAS total and subscale scores, and several GSRS domains (bloating, constipation, diarrhea, and satiety) remained statistically significant following FDR adjustment (qs < 0.05). Associations with EDE-Q7 restraint, GAD-2, and PHQ-2 did not meet FDR-adjusted significance thresholds.

Effect sizes were calculated to quantify the magnitude of group differences. Moderate effect sizes were observed for overall gastrointestinal symptom burden (GSRS total; d = 0.52), bloating (d = 0.57), and avoidant/restrictive eating (NIAS total; d = 0.50). Small-to-moderate effects were observed for constipation (d = 0.45), satiety (d = 0.48), and perceived burden of adhering to a gluten-free diet (IGFD total; d = 0.49). Smaller effects were observed for eating disorder psychopathology, anxiety, and depression symptoms (ds = 0.24–0.38). These findings suggest that the largest group differences were concentrated in gastrointestinal and dietary burden domains.

### 3.4. Linear Regression

In multivariable linear regression adjusting for age and sex, FI risk remained significantly associated with higher GSRS Total scores (β = 0.54, 95% CI [0.22, 0.86], *p* = 0.001). Age and sex were not independently associated with GSRS Total in the adjusted model. These findings suggest that the association between FI risk and gastrointestinal symptom burden persists after accounting for basic demographic factors.

## 4. Discussion

The present study examined the association between high-risk FI and a range of gastrointestinal, psychological, and dietary outcomes among individuals with CD. These findings highlight significant group differences in gastrointestinal and dietary burden between adults with CD who screened positive for risk of FI and those who were food secure.

Our findings revealed that high-risk FI was significantly associated with elevated gastrointestinal symptom severity, specifically bloating, constipation, and diarrhea. Importantly, these differences emerged even when participants shared the common experience of requiring a GFD, highlighting FI as an additional barrier to health management in CD. The magnitude of observed effects was greatest for gastrointestinal symptom burden and dietary impact, suggesting that risk of FI may be particularly relevant to the physical and practical challenges of disease management in celiac disease. The association between FI risk and overall gastrointestinal symptom burden remained significant after adjustment for age and sex, suggesting that the observed relationship is not solely attributable to demographic differences.

Adhering to a strict GFD is one of the primary ways in which people with CD manage their symptoms, yet it is also well documented as a costly and socially challenging dietary regimen [52]. The current findings suggest that FI risk was associated with greater reported challenges. Participants screening positive for risk of FI reported higher levels of gastrointestinal symptoms. Although we did not assess dietary intake patterns or objective measures of gluten exposure, the observed associations may reflect differences in food access, dietary patterns, or other unmeasured contextual factors [53,54].

It is important to note that, in individuals with CD, some restrictive eating behaviors may reflect adaptive adherence to a medically necessary gluten-free diet rather than pathological restriction. As such, elevated NIAS scores in this population may partially capture appropriate dietary vigilance. Interpretation of restrictive eating measures in medically restricted populations, therefore, warrants caution.

### 4.1. Implications for Public Health and Clinical Practice

Given the significant implications that experiencing FI has for disease management and compliance for patients with CD, it is important that every celiac patient is screened for FI. Ideally, patients will be asked the initial screening questions followed by a conversation focusing on food stability during subsequent consultation. The screening process, continuing into the consultation, provides the opportunity for the provider to gain more insight while showing compassion and empathy, which has been proven to make patients more forthcoming about social history and concerns. Additionally, this screening process enables the provider to note the social history in the patient’s record, providing access to this important information to the entire care team [55].

### 4.2. Policy and Program Recommendations

Although the present study did not assess participants’ utilization of food assistance programs (e.g., SNAP benefits or food pantries), the observed association between risk of FI and greater gastrointestinal and dietary burden highlights the potential value of structural supports. Healthcare systems may consider exploring partnerships with community-based organizations or nonprofit agencies that provide food assistance, particularly those able to accommodate medically necessary dietary restrictions such as gluten-free diets. Such partnerships represent potential avenues for intervention and warrant future study to determine feasibility, utilization, and impact on health outcomes.

### 4.3. Limitations

There are several limitations. First, although this study included a relatively large sample of individuals with CD, the subgroup of participants at high-risk of FI was modest and could limit the generalizability of findings to the broader CD population. Diagnosis was based on clinical documentation, and the dataset did not include systematic verification of biopsy status, serologic markers, or duration of gluten-free diet adherence. The absence of these measures limits inference regarding the pathway linking food insecurity risk and symptom burden. Additionally, comorbid gastrointestinal and psychiatric conditions were not assessed as exclusion criteria, which may introduce heterogeneity into the sample. The absence of this data limits our ability to determine whether the observed association between FI and gastrointestinal symptom burden reflects differences in underlying disease severity, adherence patterns, or co-occurring conditions. As such, residual confounding may partially account for the relationship between FI and symptom burden observed in this study.

Importantly, participants were adult patients who had the resources and capacity to attend in-person clinic visits at a tertiary academic medical center in an urban setting. This recruitment context may have systematically excluded individuals experiencing greater structural barriers to care, including those living in rural or medically underserved areas, individuals with limited transportation, inflexible work schedules, or fewer financial resources. 

The cross-sectional design precludes the determination of temporal directionality or causality between FI risk and gastrointestinal symptom burden. Although we observed significant associations, the analytic approach relied primarily on unadjusted group comparisons and did not incorporate comprehensive socioeconomic covariates. As such, it remains unclear whether FI risk is associated with greater symptom burden, whether greater symptom burden increases vulnerability to FI through financial strain or reduced work productivity, or whether both are influenced by shared structural determinants such as poverty, healthcare access, or broader socioeconomic disadvantage. Without accounting for income, education, employment stability, insurance coverage, or geographic food access, we cannot disentangle whether observed symptom differences reflect FI itself or upstream economic and structural factors. The present data, therefore, cannot distinguish among these competing explanations.

Additionally, although demographic data were available, the sample was predominantly White and female, limiting our ability to examine racial or ethnic disparities in FI risk within this population. Because FI is embedded within systems of structural inequality, future research with more diverse samples is needed to determine whether associations differ across sociodemographic subgroups. Prior research consistently shows that Black and Hispanic households are disproportionately affected by FI and are more likely to experience adverse health outcomes as a result [56,57]. Moreover, women, particularly women of color, are at heightened risk of negative health outcomes associated with FI due to intersecting social and structural disadvantages [58,59]. Without this information, our study cannot assess whether FI exerts differential effects across sociodemographic subgroups, which is an important consideration for developing interventions. 

Participants were recruited from a tertiary celiac disease specialty clinic, which may introduce selection bias. Individuals able to access specialty care may have greater healthcare access, financial resources, or social support compared to those managed in community or resource-limited settings. Consistent with this possibility, the prevalence of positive FI screens in our sample (15.6%) was lower than recent national estimates of household FI in the United States [55]. As such, this sample may underrepresent individuals experiencing the greatest structural barriers, and findings may not generalize to all adults with celiac disease, particularly those without access to specialty care. Nevertheless, the observed associations suggest that FI screening remains clinically relevant even within tertiary care populations.

Critically, orthorexic tendencies may be particularly relevant in medically restricted populations such as individuals with celiac disease. These can be defined by an excessive preoccupation with consuming foods perceived as “pure,” “safe,” or “healthy” [59]. Strict adherence to a gluten-free diet necessitates vigilance regarding ingredients, cross-contamination, and food sourcing. As such, elevated restrictive eating scores in this sample may reflect a continuum ranging from adaptive dietary management to maladaptive or excessive preoccupation [59]. The present study did not include measures specifically designed to assess orthorexic symptoms and, therefore, cannot distinguish between appropriate dietary vigilance and clinically significant orthorexic tendencies. Future research should incorporate validated measures of orthorexia to clarify the psychological implications of restrictive eating patterns in celiac disease.

Finally, the use of a dichotomous screening threshold did not allow for examination of gradients in FI severity. Future studies with larger samples would benefit from evaluating dose–response relationships across varying levels of FI risk.

### 4.4. Future Directions

While this study contributes valuable insights into the intersection of FI and CD, several opportunities remain for future research to deepen our understanding and improve the management of FI in individuals with CD. Future research should explore additional barriers that exacerbate FI and hinder adherence to a GFD in individuals with CD.

While this study demonstrated a significant association between FI and gastrointestinal symptom severity, it remains unclear whether this is due to intentional or unintentional gluten ingestion or other factors. Future studies looking at the presence of gluten peptides in stool or urine would be helpful in delineating this issue. Investigating other factors could uncover nuanced barriers that prevent effective GFD adherence, even in the presence of health interventions. In addition, there is a critical need for research that evaluates the effectiveness of interventions designed to address FI in individuals with CD. In contrast to prior literature demonstrating associations between FI and anxiety or depressive symptoms, we did not observe statistically significant differences in GAD-2 or PHQ-2 scores between groups. Effect sizes for these comparisons were small, suggesting that psychological distress differences, if present, were modest in magnitude within this sample. Several factors may account for this pattern. First, the use of brief screening instruments may have limited sensitivity to detect more nuanced differences in symptom severity. Second, participants were drawn from a tertiary specialty clinic and may already have been receiving medical or psychosocial care, potentially attenuating observable differences. Finally, it is possible that, within this clinical population, FI risk may be more proximally related to gastrointestinal and dietary management burden than to global anxiety or depressive symptoms. These findings underscore the need for further investigation using more comprehensive mental health assessments.

Studies should focus on how food assistance programs, such as those providing affordable, safe gluten-free foods, impact GFD adherence and health outcomes in patients with CD who are at high risk of FI. Investigating the role of community-based solutions, such as local food banks offering gluten-free options or nutrition education programs, could also reveal practical interventions that reduce the burden of FI. Research that evaluates the long-term effects of these interventions will be essential to determine their feasibility and impact. Another avenue for future research is the exploration of structural inequalities that amplify the health impacts of FI. Understanding the socio-political and economic forces at play could inform more targeted interventions that address the root causes of FI among these groups. Finally, longitudinal studies are needed to assess the long-term impacts of FI on disease progression and quality of life in individuals with CD. Tracking individuals over time would allow for a better understanding of how persistent FI influences chronic disease management and how early intervention may alter disease trajectories. 

## 5. Conclusions

This cross-sectional study examined associations between FI risk and gastrointestinal, dietary, and psychological outcomes among adults with CD. Individuals who screened positive for risk of FI reported greater gastrointestinal symptom burden and greater perceived burden associated with adherence to a gluten-free diet compared to food-secure participants. Risk of FI was also associated with higher levels of avoidant/restrictive eating symptoms.

Because of the cross-sectional design, these findings do not establish temporal directionality or causality. The observed associations may reflect differences in dietary access, disease management experiences, psychosocial stressors, or other unmeasured factors. Nevertheless, the results indicate that FI risk is associated with clinically relevant differences in symptoms and dietary burden within this tertiary care population.

Healthcare providers should screen for FI during routine clinical visits, particularly among patients with CD, as it may influence disease management and treatment adherence. Providers can offer referrals to food assistance programs or community resources that specifically cater to individuals with dietary restrictions. Additionally, educating patients about accessible, affordable, gluten-free food options may reduce barriers to adherence. 

Addressing FI is crucial for improving the health and well-being of individuals with CD. The challenges of adhering to a GFD are already difficult for many individuals, and FI only exacerbates these challenges. By identifying and addressing the barriers that FI creates, we can improve dietary adherence, reduce gastrointestinal symptoms, and enhance quality of life for individuals with CD.

## Figures and Tables

**Table 1:** Descriptive statistics among food-secure and high-risk FI participants.

Variable	Food-Secure M (SD) *N =* 315	High-Risk of FI M (SD) *N* = 62	Statistic	*p*	Cohen’s *d*
*Demographics*					
Age (years)	41.77 (17.42)	37.67 (15.65)	*t* = 1.85	0.068	0.24
**Sex, n (%)**			χ^2^ = 0.62	0.433	–
Female	241 (76.8)	51 (82.3)			
Male	73 (23.2)	11 (17.7)			
**Race, n (%)**					
White	307 (97.5)	59 (95.2)			
Asian	2 (0.6)	1 (1.6)			
Black/African American	0 (0.0)	1 (1.6)			
Hispanic/Latino	2 (0.6)	0 (0.0)			
Middle Eastern/North African	1 (0.3)	0 (0.0)			
American Indian/Alaskan Native	1 (0.3)	0 (0.0)			
Other	2 (0.6)	1 (1.6)			

***Note***. Values for age are presented as mean (SD). Cohen’s *d* is reported for age. Categorical variables were analyzed using χ^2^ tests. FI = food insecurity. Percentages are column percentages. Several race categories contain small cell counts. M = mean, SD = standard deviation.

**Table 2:** Group comparisons between food-secure and high-risk of FI participants.

Clinical Variables (Score Range)	Food-Secure M (SD) *N* = 315	High-Risk of FI M (SD) *N* = 62	*t*	*p*	FDR q	Cohen’s *d*
*Gastrointestinal Symptoms* (GSRS; 1–7)						
gsrs_pain	2.81 (1.39)	3.09 (1.31)	−1.52	0.132	0.172	0.19
gsrs_bloating	2.70 (1.39)	3.47 (1.67)	−3.41	0.001 **	0.006 **	0.57
gsrs_constipation	2.10 (1.44)	2.72 (1.49)	−2.98	0.004 **	0.009 *	0.45
gsrs_diarrhea	2.54 (1.37)	3.02 (1.53)	−2.29	0.025 *	0.043 *	0.34
gsrs_satiety	2.37 (1.58)	3.09 (1.88)	−2.84	0.006 **	0.012 *	0.48
gsrs_total	2.50 (1.14)	3.08 (1.27)	−3.29	0.001 **	0.006 **	0.52
*Impact of Gluten-Free Diet* (IGFD; 1–5)						
igfd_total	2.29 (0.93)	2.76 (1.02)	−3.42	<0.001 ***	0.006 **	0.49
*Nine Item ARFID Screener*						
nias_picky (0–15)	3.86 (3.82)	5.50 (4.80)	−2.54	0.013 *	0.025 *	0.41
nias_app (0–15)	4.12 (4.22)	6.18 (4.73)	−3.18	0.002 **	0.009 **	0.46
nias_fear (0–15)	3.90 (4.37)	5.45 (5.19)	−2.21	0.030 *	0.049 *	0.34
nias_total (0–45)	11.88 (10.08)	17.13 (12.33)	−3.15	0.002 **	0.008 **	0.50
*Eating Disorder Examination (EDEQ;* 0–6)						
edeq7_restraint	1.48 (1.99)	1.60 (2.12)	−0.41	0.680	0.680	0.09
edeq7_overval	1.98 (1.95)	2.73 (2.25)	−2.47	0.016 *	0.031 *	0.38
edeq7_bodydiss	2.37 (2.09)	3.04 (2.21)	−2.20	0.016 *	0.031 *	0.34
edeq7_global	1.94 (1.72)	2.46 (2.02)	−1.88	0.063	0.084	0.31
*Generalized Anxiety Disorder 2-Item* (GAD; 0–6)						
gad_total	2.05 (1.97)	2.45 (2.09)	−1.38	0.170	0.194	0.24
*Patient Health Questionnaire (PHQ;* 0–6)						
phq_total	1.15 (1.52)	1.45 (1.79)	−1.26	0.212	0.226	0.24

***Note***. M = mean, SD = standard deviation. Independent-samples *t-*tests were conducted, assuming unequal variances. * *p* < 0.05, ** *p* < 0.01, *** *p* < 0.001.

**Table 3:** Multivariable linear regression predicting GSRS total.

Predictor	β	SE	95% CI	*p*
FI	0.54	0.16	0.22, 0.86	0.001
Age	0.01	0.01	−0.01, 0.02	0.19
Female Sex	0.08	0.14	−0.20, 0.36	0.57

***Note***. Reference category for sex = male. β represents unstandardized regression coefficients.

## Data Availability

The datasets presented in this article are not readily available because the data are part of an ongoing study. Requests to access the datasets should be directed to hana.zickfgraf@vumc.org.

## Abbreviations

The following abbreviations are used in this manuscript:

FI	Food insecurity
CD	Celiac disease
GFD	Gluten-free diet
ARFID	Avoidance/Restrictive Food Intake Disorder

## Appendix A. Instrument Scoring Procedures

### Appendix A.1. Celiac Disease Symptom Diary and ARFID Screening Questionnaire

Binary symptom items were coded as 0 (No) and 1 (Yes). Frequency items were coded according to the numeric category selected (1–10, with ‘10+’ coded as 10). Worst abdominal pain was analyzed as a continuous 0–10 variable. Severity ratings were coded on a 1–5 scale, with higher scores indicating greater symptom severity. Items were analyzed descriptively and not combined into a composite score.

### Appendix A.2. The Gastrointestinal Symptom Rating Scale (GSRS)

Items were rated on a 7-point Likert scale (1 = Not at all to 7 = Very Severe). Subscale scores were calculated as the mean of items within abdominal pain, reflux, diarrhea, constipation, and indigestion domains. The total score was calculated as the mean of all items.

### Appendix A.3. Impact of a Gluten-Free Diet (IGFD)

Items were rated on 5-point Likert scales. A total score was calculated as the mean of all items, with higher scores indicating greater perceived dietary burden.

### Appendix A.4. Nine-Item ARFID Screener (NIAS)

Items were rated on a 6-point scale (0 = Strongly Disagree to 5 = Strongly Agree). Subscale scores (Picky Eating, Appetite/Low Interest, Fear of Aversive Consequences) were calculated by summing relevant items (range 0–15). A total score (range 0–45) was calculated by summing all items.

### Appendix A.5. Hunger Vital Signs

Responses of ‘Often true’ or ‘Sometimes true’ were coded as 1; ‘Never true’ was coded as 0. A total score ≥1 indicated screening positive for risk of food insecurity.

### Appendix A.6. Patient Health Questionnaire-2 (PHQ-2)

Items were rated 0–3 and summed (range 0–6). A score ≥3 indicates elevated risk for depressive disorder.

### Appendix A.7. Generalized Anxiety Disorder-2 (GAD-2)

Items were rated 0–3 and summed (range 0–6). A score ≥3 indicates elevated risk for anxiety disorder.

### Appendix A.8. Eating Disorder Examination Questionnaire (EDE-Q 6.0)

Behavioral frequency items were coded according to the number of days endorsed. Attitudinal items were rated 0–6. Subscale and Global scores were calculated as means of relevant items. BMI was calculated from self-reported height and weight.

## Appendix B. Strobe Checklist

**Table A1:** STROBE checklist for cross-sectional studies.

	Item	Recommendation
**Title and abstract**	1	(a) Indicated the study’s design with commonly used terms in the Title and the Abstract.
		(b) Provided an informative and balanced summary of what was done and what was found in the Abstract.
**Introduction**		
Background/rationale	2	Scientific background and rationale for the investigation reported in Section 1: Introduction.
Objectives	3	Objectives stated in Section 1: Introduction.
**Methods**		
Study design	4	Presented key elements of study design in Section 2: Methods and Materials.
Setting	5	Described the setting, locations, and relevant dates, including periods of recruitment, exposure, follow-up, and data collection in Section 2.1: Participants.
Participants	6	(a) Give the eligibility criteria and the sources and methods of selection of participants in Section 2.1: Participants.
Variables	7	Defined all outcomes, exposures, predictors, potential confounders, and effect modifiers in Section 2: Methods and Materials.
Data sources/measurement	8	For each variable of interest, gave sources of data and details of methods of assessment (measurement) in Section 2.2: Measures.
Bias	9	Described any efforts to address potential sources of bias in Section 4.3: Limitations.
Study size	10	Explained how the study size was arrived at in Section 2: Methods and Materials.
Quantitative variables	11	Explained how quantitative variables were handled in the analyses in Section 2.3: Statistical Methods.
Statistical methods	12	(a) Described all statistical methods, including those used to control for confounding in Section 2.3: Statistical Methods.
		(b) Described any methods used to examine subgroups and interactions in Section 2.3: Statistical Methods.
		(c) Explained how missing data were addressed in Section 3.2: Missing Data.
**Results**		
Participants	13	(a) Participants reported in Section 2.1: Participants.
		(b) Missingness of data reported in Section 3.2: Missing Data.
Descriptive data	14	(a) Give characteristics of study participants (eg demographic, clinical, social) and information on exposures and potential confounders in Section 3.1: Descriptive Statistics.
		(b) Indicated number of participants with missing data for each variable of interest in Section 3.2: Missing Data.
Outcome data	15	Numbers of outcome events reported in Table 1.
Main results	16	Provided unadjusted estimates and confounder-adjusted estimates and their precision (e.g., 95% confidence interval) in Section 3: Results.
		(b) Category boundaries reported when continuous variables were categorized.
Other analyses	17	Other analyses reported in Section 3: Results.
**Discussion**		
Key results	18	Key results summarized in Section 4: Discussion.
Limitations	19	Limitations of the study included in Section 4.3: Limitations.
Interpretation	20	Cautious overall interpretation of results considering objectives, limitations, multiplicity of analyses, results from similar studies, and other relevant evidence included in Section 4: Discussion.
Generalisability	21	Generalizability addressed in Section 4: Discussion.
**Other information**		
Funding	22	Funding disclosed in the funding statement.

## Appendix C. Missingness Data

**Table A2:** Proportion of missing responses by variable.

Measure/Item	% Missing Total	% Missing (FI+)	% Missing (Food-Secure)
Age (years)	1.9	1.6	1.5
Celiac diagnosis indicator (cd_dx)	0.0	0.0	0.0
GSRS item 1	15.4	4.7	16.4
GSRS item 2	15.9	4.7	17.0
GSRS item 3	17.1	7.8	17.9
GSRS item 4	15.9	4.7	17.0
GSRS item 5	15.7	4.7	16.7
GSRS item 6	15.7	4.7	16.7
GSRS item 7	15.7	4.7	16.4
GSRS item 8	15.7	4.7	16.4
GSRS item 9	15.7	4.7	16.7
GSRS item 10	15.7	4.7	16.7
GSRS item 11	15.4	4.7	16.4
GSRS item 12	15.9	4.7	16.7
GSRS item 13	15.4	4.7	16.4
IGFDQ item 1	8.3	9.4	6.3
IGFDQ item 2	8.3	9.4	6.3
IGFDQ item 3	8.1	9.4	6.0
IGFDQ item 4	8.1	9.4	6.0
IGFDQ item 5	8.6	9.4	6.6
IGFDQ item 6	8.1	9.4	6.0
IGFDQ item 7	8.3	9.4	6.3
IGFDQ item 8	8.3	9.4	6.3
IGFDQ item 9	8.6	9.4	6.6
IGFDQ item 10	8.3	9.4	6.3
IGFDQ item 11	8.3	9.4	6.3
IGFDQ item 12	8.8	9.4	6.9
IGFDQ item 13	8.1	9.4	6.0
IGFDQ item 14	8.6	9.4	6.6
IGFDQ item 15	9.0	9.4	7.2
IGFDQ item 16	9.0	12.5	6.6
IGFDQ item 17	8.8	9.4	6.6
IGFDQ item 18	8.3	9.4	6.3
NIAS item 1	2.6	0.0	0.0
NIAS item 2	2.6	0.0	0.0
NIAS item 3	2.6	0.0	0.0
NIAS item 4	2.6	0.0	0.0
NIAS item 5	2.6	0.0	0.0
NIAS item 6	2.6	0.0	0.0
NIAS item 7	2.6	0.0	0.0
NIAS item 8	2.6	0.0	0.0
NIAS item 9	2.6	0.0	0.0
EDE-Q7 item 1	4.5	0.0	0.6
EDE-Q7 item 2	4.8	1.6	0.6
EDE-Q7 item 3	4.8	0.0	0.9
EDE-Q7 item 4	4.8	0.0	0.9
EDE-Q7 item 5	4.5	0.0	0.6
EDE-Q7 item 6	4.8	0.0	0.9
EDE-Q7 item 7	4.5	0.0	0.6
GAD-2 item 1	4.3	0.0	0.0
GAD-2 item 2	4.3	0.0	0.3
PHQ-2 item 1	4.8	0.0	0.6
PHQ-2 item 2	4.8	0.0	0.6
Average across items	8.2	4.5	6.4

**Note.** Values indicate the percentage of missing responses for each variable/item. Average indicates mean missingness across listed items/variables. FI+ indicates participants screening positive for risk of food insecurity.

## References

[1] Becker C.B., Middlemass K., Taylor B., Johnson C., Gomez F. (2017). Food insecurity and eating disorder pathology. Int. J. Eat. Disord..

[2] Becker C.B., Middlemass K.M., Gomez F., Martinez-Abrego A. (2019). Eating disorder pathology among individuals living with food insecurity: A replication study. Clin. Psychol. Sci..

[3] U.S. Department of Agriculture, Economic Research Service (2025). Food Security in the U.S.: Key Statistics & Graphics. https://www.ers.usda.gov/topics/food-nutrition-assistance/food-security-in-the-us/key-statistics-graphics.

[4] Hazzard V.M., Loth K.A., Hooper L., Becker C.B. (2020). Food Insecurity and Eating Disorders: A Review of Emerging Evidence. Curr. Psychiatry Rep..

[5] Jacob L., Smith L., Kostev K., Oh H., Gyasi R.M., López Sánchez G.F., Song T.J., Tully M.A., Haro J.M., Yon D.K. (2023). Food insecurity and insomnia-related symptoms among adults from low- and middle-income countries. J. Sleep Res..

[6] Silverman J., Krieger J., Kiefer M., Hebert P., Robinson J., Nelson K. (2015). The Relationship Between Food Insecurity and Depression, Diabetes Distress and Medication Adherence Among Low-Income Patients with Poorly-Controlled Diabetes. J. Gen. Intern. Med..

[7] Troxel W.M., Haas A., Ghosh-Dastidar B., Richardson A.S., Hale L., Buysse D.J., Buman M.P., Kurka J., Dubowitz T. (2020). Food insecurity is associated with objectively measured sleep problems. Behav. Sleep Med..

[8] Trofholz A.C., Tate A., Keithahn H., de Brito J.N., Loth K., Fertig A., Berge J.M. (2021). Family meal characteristics in racially/ethnically diverse and immigrant/refugee households by household food security status: A mixed methods study. Appetite.

[9] Park S., Chen J., Bustamante A.V. (2024). Adverse Consequences of Food Insecurity Among U.S. Adults Beyond Health Outcomes. Am. J. Prev. Med..

[10] Gibert A., Espadaler M., Angel Canela M., Sánchez A., Vaqué C., Rafecas M. (2006). Consumption of gluten-free products: Should the threshold value for trace amounts of gluten be at 20, 100 or 200 p.p.m.?. Eur. J. Gastroenterol. Hepatol..

[11] Adams D.W., Moleski S., Jossen J., Tye-Din J.A. (2024). Clinical Presentation and Spectrum of Gluten Symptomatology in Celiac Disease. Gastroenterology.

[12] Ciacci C., D’Agate C., De Rosa A., Franzese C., Errichiello S., Gasperi V., Pardi A., Quagliata D., Visentini S., Greco L. (2003). Self-rated quality of life in celiac disease. Dig. Dis. Sci..

[13] Lamontagne P., West G.E., Galibois I. (2001). Quebecers with celiac disease: Analysis of dietary problems. Can. J. Diet. Pract. Res..

[14] Leinonen H., Kivelä L., Lähdeaho M.L., Huhtala H., Kaukinen K., Kurppa K. (2019). Daily Life Restrictions are Common and Associated with Health Concerns and Dietary Challenges in Adult Celiac Disease Patients Diagnosed in Childhood. Nutrients.

[15] Silvester J.A., Weiten D., Graff L.A., Walker J.R., Duerksen D.R. (2016). Living gluten-free: Adherence, knowledge, lifestyle adaptations and feelings towards a gluten-free diet. J. Hum. Nutr. Diet..

[16] Zarkadas M., Cranney A., Case S., Molloy M., Switzer C., Graham I.D., Butzner J.D., Rashid M., Warren R.E., Burrows V. (2006). The impact of a gluten-free diet on adults with coeliac disease: Results of a national survey. J. Hum. Nutr. Diet..

[17] Zarkadas M., Dubois S., MacIsaac K., Cantin I., Rashid M., Roberts K.C., La Vieille S., Godefroy S., Pulido O.M. (2013). Living with coeliac disease and a gluten-free diet: A Canadian perspective. J. Hum. Nutr. Diet..

[18] Bajaj P., Shroff P., Sansweet S., Kanaley M., Milliron C., Oleshansky E., Bajaj K., Goldman M., Weiser C., Gupta R. (2024). The pricing premium on allergen-free foods. J. Allergy Clin. Immunol..

[19] Lee A.R., Wolf R.L., Lebwohl B., Ciaccio E.J., Green P.H.R. (2019). Persistent Economic Burden of the Gluten Free Diet. Nutrients.

[20] Barratt S.M., Leeds J.S., Sanders D.S. (2011). Quality of life in Coeliac Disease is determined by perceived degree of difficulty adhering to a gluten-free diet, not the level of dietary adherence ultimately achieved. J. Gastrointestin. Liver Dis..

[21] Hallert C., Grant C., Grehn S., Grännö C., Hultén S., Midhagen G., Ström M., Svensson H., Valdimarsson T. (2002). Evidence of poor vitamin status in coeliac patients on a gluten-free diet for 10 years. Aliment. Pharmacol. Ther..

[22] Rabiee R., Mahdavi R., Shirmohammadi M., Nikniaz Z. (2024). Eating disorders, body image dissatisfaction and their association with gluten-free diet adherence among patients with celiac disease. BMC Nutr..

[23] Häuser W., Janke K.H., Klump B., Gregor M., Hinz A. (2010). Anxiety and depression in adult patients with celiac disease on a gluten-free diet. World J. Gastroenterol..

[24] Smith D.F., Gerdes L.U. (2012). Meta-analysis on anxiety and depression in adult celiac disease. Acta Psychiatr. Scand..

[25] Sharma N., Singh K., Senapati S. (2021). Celiac disease poses significant risk in developing depression, anxiety, headache, epilepsy, panic disorder, dysthymia: A meta-analysis. Indian J. Gastroenterol..

[26] Nikniaz Z., Beheshti S., Abbasalizad Farhangi M., Nikniaz L. (2021). A systematic review and meta-analysis of the prevalence and odds of eating disorders in patients with celiac disease and vice-versa. Int. J. Eat. Disord..

[27] Hedman A., Breithaupt L., Hübel C., Thornton L.M., Tillander A., Norring C., Birgegård A., Larsson H., Ludvigsson J.F., Sävendahl L. (2019). Bidirectional relationship between eating disorders and autoimmune diseases. J. Child. Psychol. Psychiatry.

[28] Wei Y., Wang Y., Yuan Y., Chen J. (2024). Celiac Disease, Gluten-Free Diet, and Eating Disorders: From Bench to Bedside. Foods.

[29] Abber S.R., Burton Murray H. (2023). Does Gluten Avoidance in Patients with Celiac Disease Increase the Risk of Developing Eating Disorders?. Dig. Dis. Sci..

[30] Gholmie Y., Lee A.R., Satherley R.M., Schebendach J., Zybert P., Green P.H.R., Lebwohl B., Wolf R. (2023). Maladaptive Food Attitudes and Behaviors in Individuals with Celiac Disease and Their Association with Quality of Life. Dig. Dis. Sci..

[31] Burton-Murray H., Sella A.C., Gydus J.E., Atkins M., Palmer L.P., Kuhnle M.C., Becker K.R., Breithaupt L.E., Brigham K.S., Aulinas A. (2024). Medical Comorbidities, Nutritional Markers, and Cardiovascular Risk Markers in Youth with ARFID. Int. J. Eat. Disord..

[32] Ma C., Singh S., Jairath V., Radulescu G., Ho S.K.M., Choi M.Y. (2022). Food Insecurity Negatively Impacts Gluten Avoidance and Nutritional Intake in Patients with Celiac Disease. J. Clin. Gastroenterol..

[33] Wang X., Anders S., Jiang Z., Bruce M., Gidrewicz D., Marcon M., Turner J.M., Mager D.R. (2024). Food insecurity impacts diet quality and adherence to the gluten-free diet in youth with celiac disease. J. Pediatr. Gastroenterol. Nutr..

[34] Crocker H., Lewis T., Violato M., Peters M. (2024). The affordability and obtainability of gluten-free foods for adults with coeliac disease following their withdrawal on prescription in England: A qualitative study. J. Hum. Nutr. Diet..

[35] Addolorato G., Capristo E., Ghittoni G., Valeri C., Mascianà R., Ancona C., Gasbarrini G. (2001). Anxiety but not depression decreases in coeliac patients after one-year gluten-free diet: A longitudinal study. Scand. J. Gastroenterol..

[36] Bruening M., Argo K., Payne-Sturges D., Laska M.N. (2017). The Struggle Is Real: A Systematic Review of Food Insecurity on Postsecondary Education Campuses. J. Acad. Nutr. Diet..

[37] Nagata J.M., Palar K., Gooding H.C., Garber A.K., Whittle H.J., Bibbins-Domingo K., Weiser S.D. (2019). Food Insecurity Is Associated with Poorer Mental Health and Sleep Outcomes in Young Adults. J. Adolesc. Health.

[38] Ashbrook A., Essel K., Montez K., Bennett-Tejes D. (2021). Screen and Intervene: A Toolkit for Pediatricians to Address Food Insecurity.

[39] Zickgraf H.F., Ellis J.M. (2018). Initial validation of the Nine Item Avoidant/Restrictive Food Intake disorder screen (NIAS): A measure of three restrictive eating patterns. Appetite.

[40] Burton Murray H., Dreier M.J., Zickgraf H.F., Becker K.R., Breithaupt L., Eddy K.T., Thomas J.J. (2021). Validation of the nine item ARFID screen (NIAS) subscales for distinguishing ARFID presentations and screening for ARFID. Int. J. Eat. Disord..

[41] Grilo C.M., Reas D.L., Hopwood C.J., Crosby R.D. (2015). Factor structure and construct validity of the Eating Disorder Examination-Questionnaire in college students: Further support for a modified brief version. Int. J. Eat. Disord..

[42] Fairburn C.G., Beglin S.J. (1994). Eating Disorder Examination Questionnaire (EDE-Q). APA PsycTests.

[43] Svedlund J., Sjödin I., Dotevall G. (1988). GSRS—A clinical rating scale for gastrointestinal symptoms in patients with irritable bowel syndrome and peptic ulcer disease. Dig. Dis. Sci..

[44] Simsek S., Baysoy G., Gencoglan S., Uluca U. (2015). Effects of Gluten-Free Diet on Quality of Life and Depression in Children with Celiac Disease. J. Pediatr. Gastroenterol. Nutr..

[45] Sapra A., Bhandari P., Sharma S., Chanpura T., Lopp L. (2020). Using Generalized Anxiety Disorder-2 (GAD-2) and GAD-7 in a Primary Care Setting. Cureus.

[46] Löwe B., Unützer J., Callahan C.M., Perkins A.J., Kroenke K. (2004). Monitoring depression treatment outcomes with the patient health questionnaire-9. Med. Care.

[47] R Core Team (2026). R: A Language and Environment for Statistical Computing.

[48] Foster K.N. (2024). Tennesseans with Children Struggle with Food Insecurity. ETSU News.

[49] Vanderbilt University Medical Center (2024). Poll: Tennessee Families with Children Say They Are Food Insecure. VUMC News.

[50] van Buuren S., Groothuis-Oudshoorn K. (2011). Mice: Multivariate imputation by chained equations in R. J. Stat. Softw..

[51] Ghunaim M., Seedi A., Alnuman D., Aljohani S., Aljuhani N., Almourai M., Alsuhaymi S. (2024). Impact of a Gluten-Free Diet in Adults with Celiac Disease: Nutritional Deficiencies and Challenges. Cureus.

[52] Leiman D.A., Madigan K., Carlin M., Cantrell S., Palakshappa D. (2022). Food Insecurity in Digestive Diseases. Gastroenterology.

[53] Madigan K., Carlin M., Cantrell S., Palakshappa D., Leiman D. (2021). Food insecurity and digestive diseases: A systematic review. Am. J. Gastroenterol..

[54] Andermann A., CLEAR Collaboration (2016). Taking action on the social determinants of health in clinical practice: A framework for health professionals. CMAJ.

[55] Coleman-Jensen A., Rabbitt M.P., Gregory C.A., Singh A. (2022). Household Food Security in the United States in 2021 (Report No. ERR-309). U.S. Department of Agriculture, Economic Research Service. https://www.ers.usda.gov/publications/pub-details?pubid=104655.

[56] Myers C.A. (2020). Food Insecurity and Psychological Distress: A Review of the Recent Literature. Curr. Nutr. Rep..

[57] Jih J., Stijacic-Cenzer I., Seligman H.K., Boscardin W.J., Nguyen T.T., Ritchie C.S. (2018). Chronic disease burden predicts food insecurity among older adults. Public Health Nutr..

[58] Laraia B.A. (2013). Food insecurity and chronic disease. Adv. Nutr..

[59] Ng Q.X., Lee D.Y.X., Yau C.E., Han M.X., Liew J.J.L., Teoh S.E., Ong C., Yaow C.Y.L., Chee K.T. (2024). On Orthorexia Nervosa: A Systematic Review of Reviews. Psychopathology.

